# A Descriptive Morphometric Approach of the Skull and Mandible of the Common Opossum *(Didelphis Marsupialis Linnaeus, 1758)* in the Caribbean and its Clinical Application during Regional Anaesthesia

**DOI:** 10.3390/vetsci5010029

**Published:** 2018-03-09

**Authors:** Reda Mohamed

**Affiliations:** 1 Department of Basic Veterinary Sciences, School of Veterinary Medicine, Faculty of Medical Sciences, The University of the West Indies, St. Augustine, Trinidad and Tobago; reda.mohamed@sta.uwi.edu; Tel.: +645-3232-4206; 2 Anatomy and Embryology Department, Faculty of Veterinary Medicine, Beni-Suef University, Beni-Suef 62511, Egypt

**Keywords:** morphometric, skull, Common opossum, Caribbean, regional anaesthesia

## Abstract

The aim of this study was to determine the morphometric values of the skull and the mandible of the common opossum from the Caribbean island of Trinidad and Tobago. The skulls and mandibles were obtained from ten opossums captured for research purposes. The skulls and mandibles were prepared and cleaned using standard method. Some anatomical landmarks of the skulls and mandibles were identified and measured. The results were important for identification of the common opossum via comparison and discussion of our results with that of other marsupial species. Furthermore, the results had clinical importance with regard to regional nerve blocks of the infraorbital, inferior alveolar, and mental nerves for dental extraction and head surgery. This study concluded that by using the anatomical landmarks of the infraorbital and mental foramina it will be easier for the veterinarian surgeons during the application of local anesthetic agent for the infraorbital, inferior alveolar, and mental nerve blocks.

## 1. Introduction

The genus Didelphis has six species of marsupials that are found only in the Americas, which are Didelphis marsupialis, Didelphis aurita, Didelphis albiventris, Didelphis pernigra, Didelphis virginiana, and Didelphis imperfecta. Common opossums are found throughout much of Central and South America and the Caribbean. They are characterized by a thick coat of fur, sharp claws, long whiskers, long tail, and black ring around both eyes and large black ears. Common opossum *(Didelphis Marsupialis)* has an economic value as it sold commercially for meat in Trinidad and Tobago. Recently, due the demand of this type of meat, some people in Trinidad started to rear this animal for met production. This will lead to the need for veterinary care in the near future. The head is needed for deglutition and olfaction and it is the location of brain, eyes, teeth, tongue, lips, and nose [1]. Studies of the morphometry and clinical anatomy of the skull has been done in the horse [2], in donkey [3], in cattle [4], in camel [5], Barbados Blackbelly sheep [6], in Rasquera goat [7], in dog [8], in rabbit [9], in cat [10], in African giant rat [11], in koala, wombat and wallaby [12,13], in marsupialis, pernigra and imperfecta [14], in yak [15], and in hedgehog [16]. There was no description of the morphometry of the skull the common opossum from the Caribbean. Thus the aim of this study was to give a detailed morphometric description of the bones of the skull and mandible of the common opossum from the Caribbean island of Trinidad and Tobago, which could be used to identify the species and in clinical application during regional anaesthesia in the head.

## 2. Materials and Methods

The skulls and mandibles of ten adult common opossums were used in this study. The opossums had been killed for research purposes in the School of Veterinary Medicine, Faculty of medical sciences, University of the West Indies, Trinidad and Tobago. The heads of both sexes were collected. The heads were free from any skeletal deformities. The skulls and mandibles were cut at the occipitoatlantal joint. The heads were boiled to remove the skin and muscles then left to dry for two weeks. The skulls and mandibles were bleached in a sealed container for four days in 3% hydrogen peroxide and then left to dry for two weeks [17]. A measuring tape was used to measure the anatomical distances (Table 1). The data was analyzed using SPSS statistics for Windows Version 20.0 (IBM, Armonk, NY, USA), to get mean and standard deviation. Also, one sample *t-test* with carried out to compare the mean measurements with that of the koala [13]. Nomina Anatomia Veterinaria [18] was used to name the bones and foramina of the skull. 

## 3. Results 

The anatomical measurements were important for identification of the common opossum species and clinically for regional nerve blocks of infraorbital, mandibular alveolar, and mental nerves.

**Figure 1:**
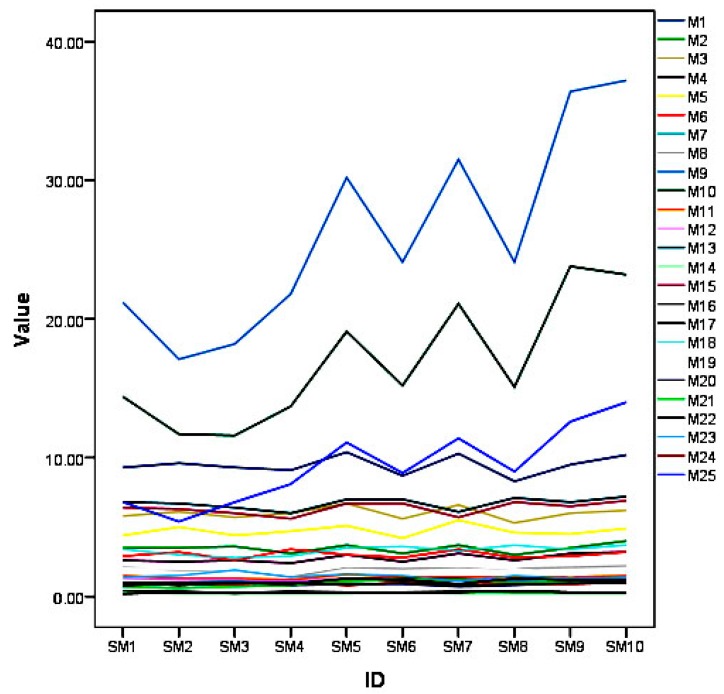
A diagram showing line graphs of each anatomical measurements of the 10 skulls and mandibles (SM) of the common opossum.

**Figure 2:**
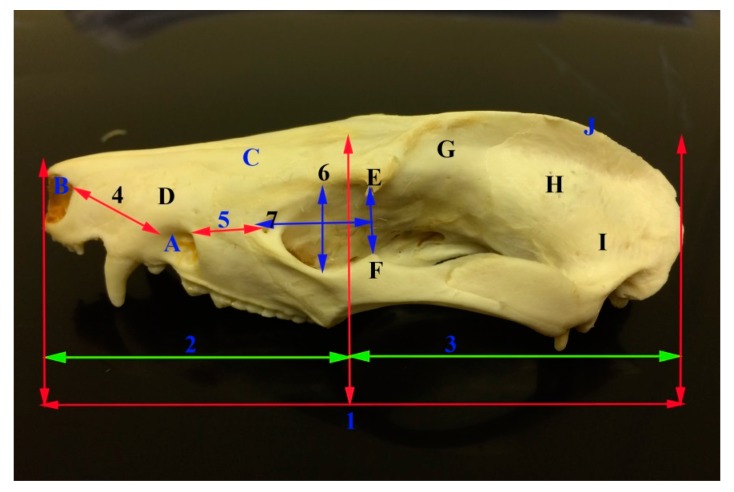
Photograph of the lateral view of the skull of the common opossum showing the skull measures. A infraorbital foramen; B a notch between the nasal and incisive bones; C nasal bone; D incisive bone; E zygomatic process of frontal bone; F frontal process of zygomatic bone; G frontal bone H Parietal bone; I temporal bone; J external sagittal crest; 1 skull length; 2 nasal length; 3 cranial length; 4 distance between the notch between the infraorbital foramen as well as nasal and incisive bones; 5 distance between infraorbital foramen and medial canthus of the eye; 6 orbital length; and, 7 orbital width.

**Figure 3:**
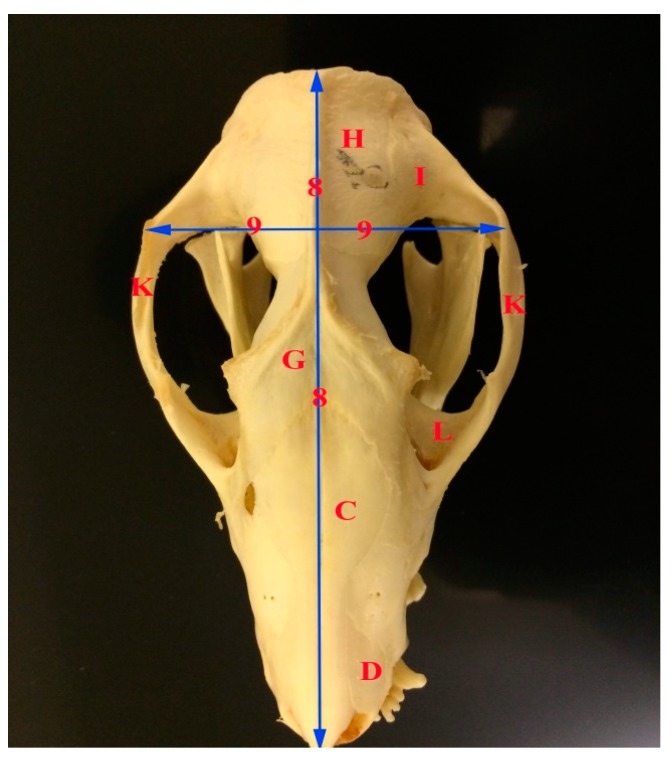
Photograph of dorsoventral view of the skull of the common opossum showing the skull measures. C nasal bone; D incisive bone; G frontal bone H Parietal bone; I temporal bone; K zygomatic arch; L orbit; 8 facial length; and, 9 facial width.

**Figure 4:**
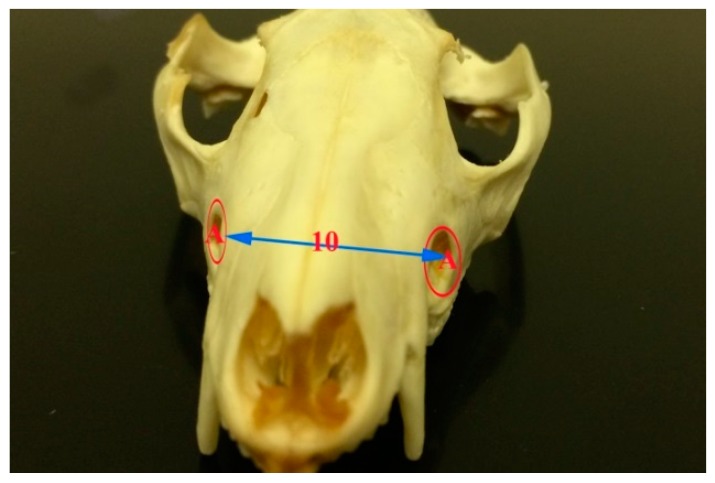
Photograph of frontal view of the skull of the common opossum showing the infraorbital foramina. A infraorbital foramen; and, 10 distance between the two infraorbital foramina.

The skull length of the common opossum was 9.47 ± 0.69 cm. The facial length was 3.47 ± 0.32 cm, while the cranial length was 6 ± 0.43 cm. The skull width was 4.73 ± 0.39 cm. The facial width was 3.02 ± 0 cm. Infraorbital foramina distance was 2.76 ± 0.30 cm. The distance between the medial canthus to the infraorbital foramen was 1.16 ± 0.14 cm. The distance between the notch between the nasal and incisive bones to the infraorbital foramen 1.98 ± 0.23 cm. Skull weight with mandible was 26.18 ± 7.22 cm, while the skull weight without mandible was 16.79 ± 4.47 cm. The orbital length was 1.4 ± 0.12 cm, while the orbital width was 1.22 ± 0.06 cm. Moreover, the skull index was 50.0 ± 2.93 cm, whereas the facial index was 87.57 ± 10.23 cm. The orbital indices were 87.37 ± 3.60 cm (Figure 1, Figure 2, Figure 3 and Figure 4, Table 2).

**Figure 5:**
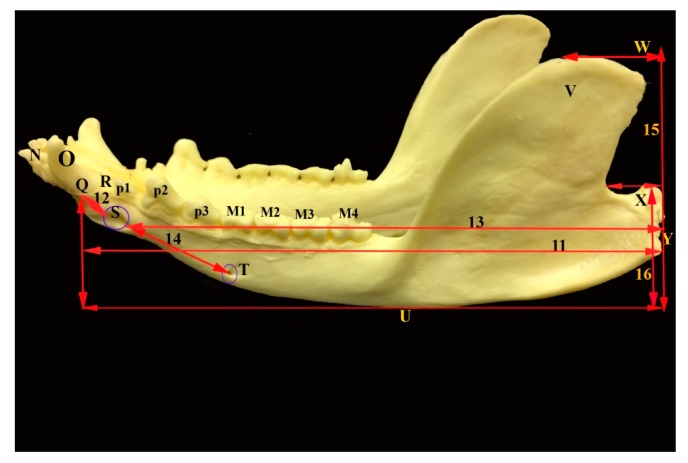
Photograph of lateral view of the mandible of the common opossum showing the mandible measures. M (1–4) lower molar teeth; N incisor teeth; O canine; P (1–3) lower premolar teeth; Q lateral alveolar border; R lateral alveolar border of first lower premolar tooth; S rostral mental foramen; T caudal mental foramen; U base of mandible; V coronoid process; W highest point of the coronoid process; X condylar process; Y caudal border of the mandible; 11 mandibular length; 12 distance between lateral alveolar border and rostral mental foramen; 13 distance between the caudal border of the mandible to the caudal mental foramen; 14 distance between the rostral and caudal mental foramina; 15 maximum mandibular height; and, 16 distance between the condyloid fossa to the base of the mandible.

**Figure 6:**
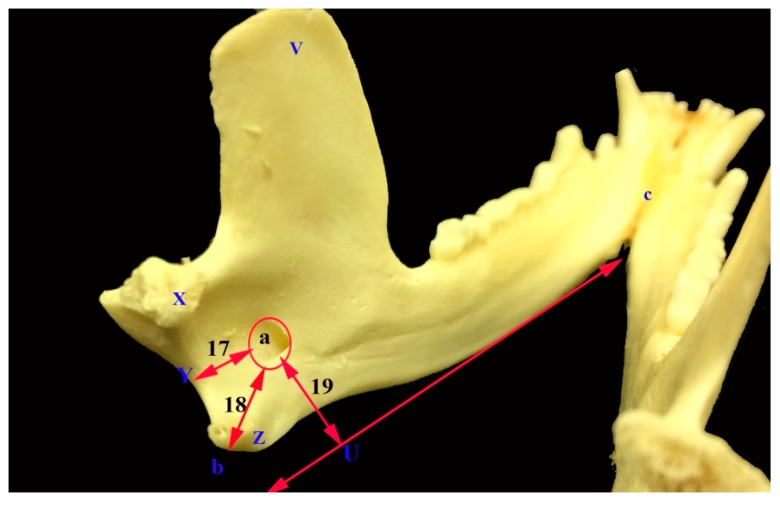
Photograph of caudal view of the mandible of the common opossum showing the mandibular foramen and its anatomical landmark parameters. V coronoid process; X condylar process; Z angular process; U base of the mandible; Y caudal border of the mandibular foramen; a mandibular foramen; b mandibular angle; and, c mandibular symphysis.

The mandible was the largest bone of the skull. The left and right mandibles were united rostrally at the mandibular symphysis. The mandible length in opossum was 6.71 ± 0.41 cm and the mandible height was 3.33 ± 0.33 cm. The mandibular weight was 9.41 ± 2.79 gm. Furthermore, the distance between the lateral alveolar border of the first lower premolar to the rostral mental foramen was 0.22 ± 0.04 cm. The distance between the caudal mandibular border to the rostral mental foramen was 6.36 ± 0.460 cm. The distance between the lateral alveolar to the rostral mental foramen was 33 ± 0.07 cm. The distance between the condyloid fossa to base of the mandible was 1.96 ± 0.24 cm. The distance between the caudal border of the mandible to the mandibular foramen was 0.82 ± 0.27 cm. While, the distance between the base of the mandible to the mandibular foramen was 0.94 ± 0.22 cm and the distance between the mandibular angle to the mandibular foramen was 1.08 ± 0.18 cm. The distance between the caudal and rostral mental foramina was 1.46 ± 0.21 and between the rostral mental foramen and incisor root was 0.9 ± 0.08 cm (Figure 1, Figure 5 and Figure 6, Table 2).

The infraorbital canals were formed by the infraorbital foramina cranially and maxillary foramina caudally. The infraorbital foramen was detected by calculating the distances between this foramen and either medial canthus of the eye or rostral edge of the nasal bone. The infraorbital canal acted as the passages for the maxillary branches of the trigeminal nerves, as well as the infraorbital arteries and veins. The maxillary nerve entered the maxilla through the maxillary foramen. The nerve gave off alveolar branches to the cheek teeth within the infraorbital canal then exited from the infraorbital foramen as infraorbital nerve which innervate the skin of the lateral nasal region, mucosa and skin of the nasal vestibule, and the skin of the maxillary lip. To block this nerve, a needle could be inserted into either the maxillary foramen or the infraorbital foramen. It will be easier to desensitize the nerve within the infraorbital canal and its exit via the infraorbital foramen and the local anesthetic drug progressed caudally in the infraorbital canal by pressure.

There were mandibular foramina on the medial surfaces of the two rami of the mandible, near the angular processes. The mandibular foramen was detected by calculating the distances between it and either the mandibular angle, the caudal border of the mandible or the base of the mandible. The mandibular and mental foramina formed the mandibular canal for the passage of the inferior alveolar nerves of the mandibular division of the trigeminal nerve. The inferior alveolar nerve passed through the mandibular foramen. It innervated the skin of the lips and chin, gum and mandibular teeth with their alveoli. The inferior alveolar nerve could be blocked either via the mandibular or mental foramina. The blocking of the nerve to desensitize the lower teeth and lip by inserting the drug by a needle into the mandibular foramen could be difficult, causing hemorrhage due to blood vessels injuries. However the needle could be directed in a rostro-caudal direction via the rostral mental foramen as an easier way.

There were two mental foramina on each side of the body of the mandible laterally. The rostral mental foramen lied in front of the first lower premolar (P1), while the caudal one lied under the second lower molar (M2). The location of the rostral mental foramen was detected by measuring the distances between this foramen and either the caudal border of the mandible, lateral alveolar border of the mandible, the ventral border of the mandible, or the lateral alveolar border of the first premolar tooth. The inferior alveolar nerve emerged from the mental foramen as the mental nerve. The latter nerve innervated the lower lip and chin. Blocking of the mental nerve could be occurred close to its exit from the rostral mental foramen in front of the lower premolar.

## 4. Discussion

There were many morphometric studies were published in marsupial species but there were no morphometric studies on the skull of the common opossum (manicou) from the Caribbean island of Trinidad and Tobago. The results of the present study were compared with that of the others in marsupials (Table 3 and Table 4). 

The current investigation showed that the skull length of the common opossum was lower that the value reported in koala, wombat, and wallaby [12], and higher that the value reported in the hedgehog [16]. Moreover, the nasal skull length was almost similar to that of koala [12] and shorter than the value reported in wombat and wallaby [12] and Marsupialis, pernigra imperfecta [14]. The cranial skull length was lower than the value mentioned in koala, wombat, and wallaby [12].

The distances between the two infraorbital foramina, as well as between the infraorbital foramen and the medial canthus of the eye and the notch between the nasal bone and incisive bones were 2.76 ± 0.30 cm, 1.16 ± 0.14 cm, and 1.98 ± 0.23 cm, respectively. Values are not given for other marsupial species.

The current investigation showed that the skull and facial indices of the common opossum was lower than the value that was reported in Koala, wombat, and wallaby [12], and in hedgehog [16]. Moreover the orbital index value was higher than the value mentioned in wombat and higher than that of Koala and wallaby [12].

The morphometry and optometry of the skull of the common opossum from the Caribbean was useful for useful for comparative anatomical and developmental studies as well as importance in paleontological studies of the marsupialis species [12,19].

The mandible was the largest bone of the skull. The left and right mandibles were rostrally united at the mandibular symphysis. While, the unfused mandible is present in koala and goat [13,20].

The mandibular length and the maximum mandibular height of the opossum were lower than the values obtained in the koala, wombat, and wallaby [13], and in marsupialis, pernigra imperfecta [14], while the mandibular length was higher that the value reported in the hedgehog [16]. The obtained results showed that the distance from the base of the mandible to the condyloid process was lower than in the koala, wombat, and wallaby [13].

The obtained results showed that the distance from the mandibular foramen to the caudal border of the mandible was lower than the values obtained in the koala, wombat, and wallaby [13]. Also, the distance of the mandibular foramen to the base of the mandible in the opossum was lower than the values obtained in the koala and wombat, while it was higher than the value obtained in the wallaby [12]. Further, the results showed that the distance from the mandibular angle to the mandibular foramen was nearly equal to what was observed in wallaby, while it was lower than the value reported in the koala and wombat [13].

The results of the present study were similar to [21] that the anatomical measurements of the mandibular foramen were of clinical importance for the blocking the inferior alveolar nerve for desensitization of the lower jaw, lips, and teeth. In the present study, there was extra mental foramen; similar result was recorded in wombat [13], in dog [22], and in one-humped camel [6].Also The results showed that the distance between the lateral alveolar root and the mental foramen and the distance between the caudal mandibular border to the mental foramen was 0.22 ± 0.04 cm and 6.36 ± 0.46 cm, respectively. Values are not given for other marsupial species.

The obtained results, as well as those [21], stated that anatomical measurements of the mental foramen were vital as it could be used as easier way for the blocking of the infra alveolar nerve by injection of local anesthetic drugs for desensitization of lower jaw with its teeth and lower lips through the rostral mental foramen. 

The results showed that all of the measurements of the common opossum were significantly smaller than the other marsupial species, namely Koala, Wombat, and Wallaby, except for the nasal length of the skull. This can aid in the identification of the particular species.

## 5. Conclusions

The morphometric values of the skull and mandible and their clinical anatomy were important for identification of the common opossum species from the Caribbean and for comparative anatomy of other marsupial species. Furthermore, as the opossum is used as a source of meat production in Trinidad and Tobago. Recently persons started to rear this animal for economic gain, which will lead to increase the need for veterinary care, so the results will help the veterinarian surgeons to use the easier ways for the infraorbital, inferior alveolar, and mental nerve block during the application of surgical operations in the head region and during dental extraction via the injection of the anesthetic drug through the infraorbital and mental foramina. So, that the surgical procedures could be performed using regional anaesthesia, and this will allow for shorter surgical time, less anesthetic equipment, and low cost of the procedure.

## Figures and Tables

**Table 1:** Anatomical measurements of the skull and mandible of the common opossum.

Item	Measures	Item	Measures
1	Skull length	15	Caudal mandibular border to mental foramen
2	Nasal skull length (facial length)	16	Lateral alveolar border to mental foramen
3	Cranial skull length	17	Ventral border of the mandible to mental foramen
4	Infraorbital foramina distance	18	Maximum mandibular height
5	Skull width	19	Condyloid process to base of the mandible height
6	Facial width	20	Caudal border of the mandible to mandibular foramen
7	Medial canthus to the infraorbital foramen	21	Base of the mandible to mandibular foramen
8	Rostral edge of the nasal bone to the infraorbital foramen	22	Mandibular angle to mandibular foramen
9	Skull weight with mandible	23	Distance between the caudal and rostral mental foramen
10	Skull weight without mandible	24	Distance between the rostral mental foramen and incisor root
11	Orbital length	25	Mandible weight
12	Orbital width	26	Facial index: Facial width/Facial length ×100
13	Mandibular length	27	Orbital index: Orbital width/Orbital length ×100
14	Lateral alveolar border of the first lower premolar to mental foramen	28	Skull/cephalic index: Skull width/Skull length ×100

**Table 2:** Morphometry of the skull and mandible (SM) of the common opossum.

Item	SM1	SM2	SM3	SM4	SM5	SM6	SM7	SM8	SM9	SM10	Min.	Max.	Mean	± SD
1	9.30	9.60	9.30	9.10	10.40	8.70	10.30	8.30	9.50	10.20	8.30	10.40	9.47	0.69
2	3.50	3.50	3.60	3.10	3.70	3.10	3.70	3.00	3.50	4.00	3.00	4.00	3.47	0.32
3	5.80	6.10	5.70	6.00	6.70	5.60	6.60	5.30	6.00	6.20	5.30	6.70	6.00	0.43
4	2.60	2.50	2.60	2.40	3.00	2.50	3.10	2.60	3.10	3.20	2.40	3.20	2.76	0.30
5	4.40	5.00	4.40	4.70	5.10	4.20	5.50	4.60	4.50	4.90	4.20	5.50	4.73	0.39
6	2.90	3.20	2.60	3.40	3.00	2.80	3.40	2.80	2.90	3.20	2.60	3.40	3.02	0.27
7	1.00	1.00	1.10	1.00	1.20	1.30	1.30	1.10	1.40	1.20	1.00	1.40	1.16	0.14
8	2.10	1.90	1.90	1.40	2.10	2.00	2.10	2.00	2.10	2.20	1.40	2.20	1.98	0.23
9	21.20	17.10	18.20	21.80	30.20	24.10	31.50	24.10	36.40	37.20	17.10	37.20	26.18	7.22
10	14.40	11.70	11.60	13.70	19.10	15.20	20.10	15.10	23.80	23.20	11.60	23.80	16.79	4.47
11	1.50	1.30	1.30	1.20	1.60	1.40	1.40	1.40	1.40	1.50	1.20	1.60	1.40	0.12
12	1.30	1.20	1.20	1.10	1.30	1.20	1.20	1.20	1.20	1.30	1.10	1.30	1.22	0.06
13	6.80	6.70	6.40	6.00	7.00	7.00	6.10	7.10	6.80	7.20	6.00	7.20	6.71	0.41
14	0.30	0.20	0.20	0.20	0.20	0.30	0.20	0.20	0.20	0.20	0.20	0.30	0.22	0.04
15	6.40	6.30	6.00	5.60	6.70	6.70	5.70	6.80	6.50	6.90	5.60	6.90	6.36	0.46
16	0.40	0.40	0.20	0.40	0.30	0.30	0.40	0.30	0.30	0.30	0.20	0.40	0.33	0.07
17	0.20	0.30	0.30	0.30	0.30	0.30	0.30	0.40	0.30	0.30	0.20	0.40	0.30	0.05
18	3.40	3.00	2.80	2.90	3.50	3.60	3.30	3.70	3.40	3.70	2.80	3.70	3.33	0.33
19	2.00	2.00	1.90	1.60	2.20	2.20	2.20	2.00	1.50	2.00	1.50	2.20	1.96	0.24
20	1.00	1.00	1.00	0.80	0.90	0.90	0.70	0.80	1.00	1.00	0.70	1.00	0.92	0.11
21	0.70	0.60	0.70	0.80	1.10	1.20	1.20	1.00	1.10	1.00	0.60	1.20	0.94	0.22
22	1.00	0.80	1.00	0.90	1.30	1.20	0.90	1.30	1.20	1.20	0.80	1.30	1.08	0.18
23	1.40	1.50	1.90	1.40	1.60	1.50	1.10	1.50	1.30	1.40	1.10	1.90	1.46	0.21
24	0.80	0.90	0.90	1.00	0.80	1.00	0.80	0.90	0.90	1.00	0.80	1.00	0.90	0.08
25	6.80	5.40	6.80	8.10	11.10	8.90	11.40	9.00	12.60	14.00	5.40	14.00	9.41	2.79
26	82.90	91.40	72.20	109.70	81.10	90.30	91.90	93.30	82.90	80.00	72.20	109.70	87.57	10.23
27	86.70	92.30	92.30	91.70	81.30	85.70	85.70	85.70	85.70	86.70	81.30	92.30	87.37	3.60
28	47.30	52.10	47.30	51.60	49.30	48.30	53.40	55.40	47.40	48.00	47.30	55.4	50.00	2.93

**Table 3:** Comparison of the morphometry the skull and mandible of the common opossum and other marsupial species.

Parameter	Common Opossum	Koala	Wombat	Wallaby	Marsupialis	Pernigra	Imperfecta	Hedgehog
1	9.47 ± 0.69	12.0 ± 0.9	16.6 ± 0.8	13.2 ± 1.4				4.08 ± 0.11
2	3.47 ± 0.32	3.6 ± 0.6	6.4 ± 0.6	5.6 ± 1.3	47.32 ± 3.64	47.84 ± 1.89	40.52 ± 3.59	
3	6 ± 0.43	8.7 ± 1.1	10.4 ± 0.5	7.7 ± 0.4				
4	2.76 ± 0.30							
5	4.73 ± 0.39	7.0 ± 0.6	12.9 ± 0.5	7.4 ± 0.7				
6	3.02 ± 0.27	6.7 ± 0.5	9.5 ± 3.0	6.4 ± 0.5				
7	1.16 ± 0.14							
8	1.98 ± 0.23							
9	26.18 ± 7.22	70.3 ± 10.4	361.5 ± 81.3	90.7 ± 23.1				
10	16.79 ± 4.47	44.3 ± 8.1	224.5 ± 50.2	55.7 ± 13.6				
11	1.4 ± 0.12							
12	1.22 ± 0.06							
13	6.71 ± 0.41	9.8 ± 5.66	12.7 ± 8.89	9.9 ± 5.72	82.32 ± 6.32	80.10 ± 3.91	72.27 ± 5.91	3.16 ± 0.07
14	0.22 ± 0.04							
15	6.36 ± 0.46							
16	0.33 ± 0.07							
17	0.3 ± 0.05							
18	3.33 ± 0.33	6.8 ± 3.39	8.05 ± 5.69	4.1 ± 2.37				
19	1.96 ± 0.24	5.7 ± 3.29	6.75 ± 4.78	4.2 ± 2.42				
20	0.82 ± 0.27	1.3 ± 0.75	2.1 ± 1.48	2.7 ± 1.56				
21	0.94 ± 0.22	2.6 ± 1.84	2.25 ± 1.50	0.8 ± 0.57				
22	1.08 ± 0.18	2.3 ± 1.63	3.05 ± 2.16	1.2 ± 0.69				
23	1.46 ± 0.21	2.4 ± 1.39						
24	0.9 ± 0.08	1.3 ± 0.75	2.2 ± 1.56	1.7 ± 0.98				
25	9.41 ± 2.79	26 ± 15.01	137 ± 96.87	35 ± 24.75				
26	87.57 ± 10.23	186.111	148.437	114.286				
27	87.37 ± 3.60	90.00	85.294	91.304				
28	50.0 ± 2.93	58.333	77.710	56.060				54.0 ± 1.12

**Table 4:** One- sample test showing the differences between the common opossum and Koala.

Parameters	Common Opossum, M ± SD (I)	Koala, M ± SD (J)	Mean Difference (I–J)	Significance
1	9.47 ± 0.69	12.0 ± 0.9	−2.53	Significant
2	3.47 ± 0.32	3.6 ± 0.6	−0.13	Not Significant
3	6 ± 0.43	8.7 ± 1.1	−2.70	Significant
5	4.73 ± 0.39	7.0 ± 0.6	−2.27	Significant
6	3.02 ± 0.27	6.7 ± 0.5	−3.68	Significant
13	6.71 ± 0.41	9.8 ± 5.66	−3.09	Significant
20	0.82 ± 0.27	1.3 ± 0.75	−0.48	Significant
24	0.9 ± 0.08	1.3 ± 0.75	−0.40	Significant
27	87.37 ± 3.60	90.00	−2.63	Significant
